# Evaluation of Automated Measurement of Hair Density Using Deep Neural Networks

**DOI:** 10.3390/s22020650

**Published:** 2022-01-14

**Authors:** Minki Kim, Sunwon Kang, Byoung-Dai Lee

**Affiliations:** 1 Department of Computer Science, Graduate School, Kyonggi University, 154-42, Gwanggyosan-ro, Yeongtong-gu, Suwon-si 16227, Gyeonggi-do, Korea; mkmk0612@kyonggi.ac.kr (M.K.); k20201101113@kyonggi.ac.kr (S.K.); 2 Division of AI & Computer Engineering, Kyonggi University, 154-42, Gwanggyosan-ro, Yeongtong-gu, Suwon-si 16227, Gyeonggi-do, Korea

**Keywords:** deep learning, follicle detection, hair density measurement, hair transplant, object detection

## Abstract

Recently, deep learning has been employed in medical image analysis for several clinical imaging methods, such as X-ray, computed tomography, magnetic resonance imaging, and pathological tissue imaging, and excellent performance has been reported. With the development of these methods, deep learning technologies have rapidly evolved in the healthcare industry related to hair loss. Hair density measurement (HDM) is a process used for detecting the severity of hair loss by counting the number of hairs present in the occipital donor region for transplantation. HDM is a typical object detection and classification problem that could benefit from deep learning. This study analyzed the accuracy of HDM by applying deep learning technology for object detection and reports the feasibility of automating HDM. The dataset for training and evaluation comprised 4492 enlarged hair scalp RGB images obtained from male hair-loss patients and the corresponding annotation data that contained the location information of the hair follicles present in the image and follicle-type information according to the number of hairs. EfficientDet, YOLOv4, and DetectoRS were used as object detection algorithms for performance comparison. The experimental results indicated that YOLOv4 had the best performance, with a mean average precision of 58.67.

## 1. Introduction

Owing to stress and dietary changes, hair loss has recently become more prevalent not only in the elderly but also in younger generations, and the overall number of hair-loss patients, both men and women, is rapidly increasing. As a result, the hair transplantation market is one of the fastest-growing healthcare specialties and the total market size for hair restoration surgery has increased 10% since 2016 (specifically, from USD 4.1 billion USD in 2016 to USD 4.6 billion USD in 2019) [1]. For hair transplantation, hairs in the occipital donor area are typically removed and transplanted to the hair-loss areas, which requires the hairs in the occipital donor area to be counted to determine the available contribution [2,3]. This hair-density measurement (HDM) process, performed manually by doctors, is time consuming and requires a high level of expertise to make an accurate diagnosis [4,5].

Although image-processing-based approaches have dominated the literature on HDM, they have limitations such as unreliable measurement for crossing or overlapping hairs [4,6] and sensitivity to configuration parameters [7]. The recent development of object detection technology using deep learning and the establishment of related large-scale datasets have enabled the method of measuring the number of hairs by detecting only the hair follicles, which are the roots of hair, rather than recognizing the entire hair [8]. Accordingly, this study was conducted to evaluate the accuracy of various deep-learning-based HDM algorithms and determine the feasibility of automating HDM. The workflow of the deep-learning-based HDM algorithms is as follows: First, the algorithm detects the locations of hair follicles present in the donor area and classifies the type of follicles detected. The follicle type is defined by the number of hairs in it. The object detection networks used for measuring the hair density in this study were EfficientDet [9], YOLOv4 [10], and DetectoRS [11], which exhibited state-of-the-art object detection performances on various benchmark datasets.

The remainder of this paper is organized as follows. Section 2 describes relevant studies on existing HDM techniques. Section 3 describes the datasets and object detection networks used in the experiments. Section 4 compares the detection and classification performance of the three object detection networks using mAP and visualizes the results. Section 5 presents the discussion, conclusion, and ruminations about future studies.

## 2. Related Work

Prior to the advent of deep learning, most automated HDMs were performed using image processing techniques [4,6,7,12]. For instance, Shih et al. [4] preprocessed input images using various techniques, such as contrast stretching [13], color morphology [14], and Otsu thresholding [15]. The preprocessed image undergoes binarization and multi-scale line detailing procedures to separate the hair and scalp, label the hairs, and count the number of hairs. Shih [7] preprocessed images through color-to-grayscale conversion and binarization to adjust the brightness and eliminate the noise via color morphology. Then, using the multi-scale line detailing technique, the hair was colored and separated from the scalp. Zhang and Eun [6] used the Otsu algorithm to separate hair and scalp, and then processed the hair differently according to the length. For instance, short hairs were directly counted, whereas long hairs were processed using the Hough transform [16] to address the problem of overlapping hairs. The method proposed by Zhang and Eun [6] measures the number of hairs by each stand of short hair and long hair separately. However, it exhibited poor performance, particularly when a hair was long and bent or multiple hairs crossed each other. Kim et al. [12] proposed a technique for measuring the hair density using a portable camera on a smartphone, in which the hairs were identified by applying various component technologies, including image preprocessing techniques such as contrast stretching and morphology processing, skeleton conversion, and line endpoint detection.

The abovementioned image-processing-based algorithms and techniques had limitations in that the hair was not detected properly, particularly when there was an overlap of hairs or foreign substances in the scalp. However, object detection algorithms based on deep neural networks overcome these shortcomings. For instance, ScalpEye [8] uses Faster R-CNN [17] and a single-shot detector [18] to effectively detect scalp diseases (e.g., dandruff, folliculitis, and hair loss). However, ScalpEye requires a larger image than those in other hair-related studies and, although the hair follicles are detected, the number of hairs present in the follicles is unknown. Jakubík et al. [5] preprocessed training and test datasets through axis conversion and rotation. They applied convolution layers, a rectified linear-unit activation function, and a pooling layer for dimension reduction for a detection model. The preprocessing method resulted in detection improvements. Furthermore, it was found that the appropriate rotation angle for the dataset affected the detection performance. Finally, Gallucci et al. [19] investigated the feasibility of automatic skin hair counting using early deep learning models such as LeNet [20] and VGG-Net [21]. Although their work was for skin images, which are less complex than hair scalp images, they also reported that the prediction error was close to that achieved by a human for skin hair counting.

This study evaluated the accuracy of hair density measurement by applying state-of-the-art object detection algorithms, which were trained by using hair scalp RGB images from real-world clinical practice. The experimental results are expected to serve as a cornerstone for assessing the effectiveness of deep-learning-based automated HDM algorithms.

## 3. Datasets and Methods

### 3.1. Datasets

**Figure 1:**
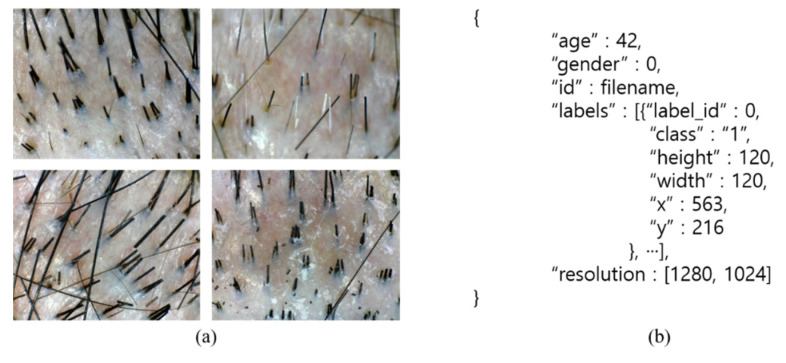
Examples of (**a**) images and (**b**) annotation information in the dataset.

In this study, we used the dataset published by the National Information Society Agency [22]. It comprised 4492 enlarged hair scalp RGB images from 817 male hair-loss patients and their corresponding annotation data. All images had a resolution of 1280 × 1024 pixels. The annotation data for each image included gender, the location and class information of hair follicles where the hair was present, and the total number of hairs in the donor area. The class of a hair follicle is determined by the number of hairs present in the hair follicle. For instance, the hair follicles with one hair, two hairs, and three hairs are classified as Classes 0, 1, and 2, respectively. As the hair follicles with four or more hairs are difficult to locate, they are classified as a single group as Class 3. Figure 1 shows an example of this; Table 1 lists the demographic information of the dataset.

The data were randomly split into three subsets without any overlap—training (60%), validation (20%), and testing (20%). During training of the object detection models, we artificially increased the size of the training and validation datasets through simple data augmentation to reduce overfitting and achieve a high classification accuracy. Specifically, a given image was vertically flipped; subsequently, both the original and flipped images were rotated by +15 and −15°, respectively. A total of 21,561 images were used for training and validation.

### 3.2. Methods

In this study, three state-of-the-art object detection models, EfficientDet, YOLOv4, and DetectoRS, were used for detecting the hair follicles in the input image and classifying their corresponding types. Subsequently, for each class, the number of follicles identified for each class was multiplied by the number of hairs per corresponding hair follicle class; finally, the obtained values were added to compute the total number of hairs in the occipital donor area. Object detection is a computer vision technique that involves identifying and locating objects within an image or video. An object detection method can be classified as a two-stage detector if region proposal and object classification are performed as separate processes. If an object detection method skips the region proposal stage and runs object detection directly over a dense sampling of possible locations, it is called a one-stage detector. All of the detection models used in this study were one-stage detectors.

EfficientDet uses a pretrained EfficientNet [23] with ImageNet [24] as a backbone network. For accurate feature extraction, EfficientDet utilizes the bi-directional feature pyramid network (BiFPN). BiFPN enhances the existing feature pyramid network (FPN) [25] by effectively aggregating the multi-scale features in a top-down manner. Furthermore, EfficientDet applies the compound scaling technique to the backbone network, feature extraction network, and prediction network, thereby successfully improving the performance. The compound scaling technique is utilized in EfficientNet; it provides a means of increasing the model capacity by simultaneously accounting for width, depth, and resolution, which are major factors that determine the model capacity and amount of computations. For HDM, the EfficientDet-D0 configuration was used.

YOLOv4 is an improved object detection network that overcomes the small object detection vulnerability of YOLO [26] by applying large input resolutions. The backbone of YOLOv4 is based on the cross-stage partial network (CSPNet) [27], which speeds up the training process with a reduced amount of computation, allowing the network to be used in any environment without performance degradation. CSPNet partitions the feature map of the base layer into two parts and merges them through a cross-stage hierarchy [27]. The use of a split and merge strategy allows for more gradient flow through the network.

Two important components of DetectoRS enabling performance boost are recursive FPN and switchable atrous convolution. The former extends the existing FPN by incorporating extra feedback connections from FPN into the bottom-up backbone layers. The latter convolves the features with different atrous rates and gathers the results using switch functions [11]. On the COCO dataset [28], DetectoRS achieved 55.7% box average precision (AP) for object detection and outperformed the state-of-the-art object detection models such as YOLOv3 [29], SpineNet [30], and Cascade R-CNN [31].

In this study, all deep learning models were built using the PyTorch framework with a CUDA back-end. For training and testing, two NVIDIA GeForce RTX 2080 super graphic cards were used in the Ubuntu 18.04.5 LTS environment. Further, to explore the generalization capability of individual deep learning models, we produced the results without any fine-tuning or modification of their official source codes except for some of the hyperparameter settings for training. In addition, as the sizes of the input images required by individual deep learning models differ (e.g., EfficientDet: 512 × 512, YOLOv4: 640 × 640, and DetectoRS: 1333 × 800), the hair scalp images were first scaled down accordingly before being fed into the deep learning models, and the final output images were scaled up to the original image size. The details of the hyperparameters used in each network are listed in Table 2.

As a performance metric, the mean average precision (mAP) was used, as it is more useful for the quantitative performance evaluation of different algorithms than precision-recall graphs. In particular, AP can be calculated over a range of Intersection over Union (IoU) thresholds. For instance, for mAP(50) the AP of a given class is calculated with IoU > 0.5, denoted AP(50). In this paper, where no distinction is made, mAP and mAP(50:95) are used interchangeably, as in other object detection studies [23,32]. In addition, recall, precision, and accuracy were measured based on the results of follicle detection. The corresponding equation is Equation (1), where N denotes the number of classes to classify and *AP_k_* denotes the average precision for class k. Furthermore, *TP*, *FP*, *TN* and *FN* denote the number of true positive, false positive, true negative, and false negative follicles, respectively.

(1) $$\mathrm{mAP}=\frac{1}{N}\overset{k=N}{\underset{k=1}{\sum }}AP_{k}\;\mathrm{precision}=\frac{TP}{TP+FP}\;\mathrm{recall}=\frac{1}{TP+FN}\;\mathrm{Accuracy}=\frac{TP+TN}{TP+TN+FP+FN}$$

## 4. Experimental Results

**Figure 2:**
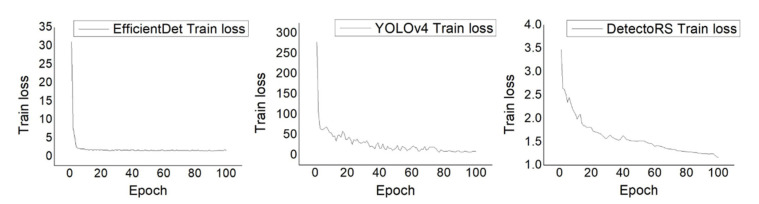
Loss curves of the training process.

Figure 2 shows the training loss during the training of the object detection models. In all cases, the curves converged properly with the hyperparameter settings, suggesting that the models had learned as much about the data as possible.

As for the hair follicles located at the edge of the image, the appearance of the hair follicles was often blurred or parts of the hair follicles were cut off, which made it difficult to classify these hair follicles with the naked eye. To increase the objectivity of the evaluation, an ellipse was drawn based on the center of the input hair RGB image, and hair follicles existing outside the circle were excluded from the evaluation.

Experimental results showed that YOLOv4 exhibited the highest detection performance among the three detection models, with a mAP of 58.67, while EfficientDet and DetectoRS showed mAPs of 31.97 and 37.22, respectively (see Table 3). While all three models detected similar areas, inaccurate classification results led to differences in performance. In addition, YOLOv4 had a lower rate of redundant detection.

**Figure 3:**
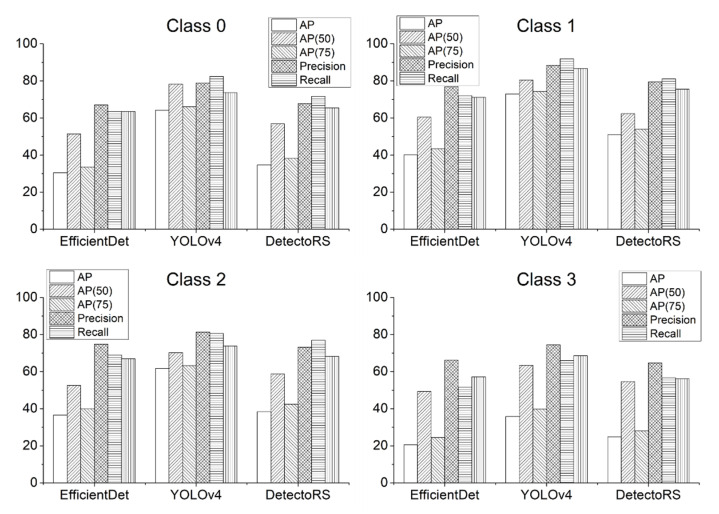
Performance comparison by class.

Figure 3 shows the comparative performance of individual deep learning models by follicle classes. For each of the hair follicle classes, YOLOv4 outperformed the other detection models. However, for hair follicles of Class 3, although YOLOv4 performed the best among them, all of the detection models showed relatively poor performance compared to the detection of other hair follicle classes. This result is attributable to the fact that it is more difficult to classify Class 3, as the hair follicles of Class 3 have the features of hair follicles of both Classes 1 and 2. In addition, class imbalance may have also been a contributing factor.

**Figure 4:**
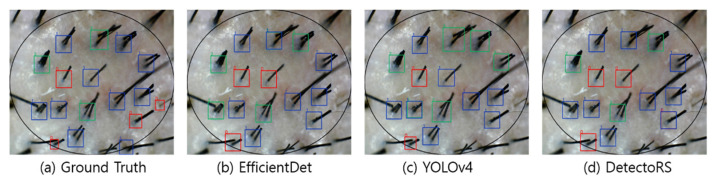
Visualization of detection results in a short hair image.

**Figure 5:**
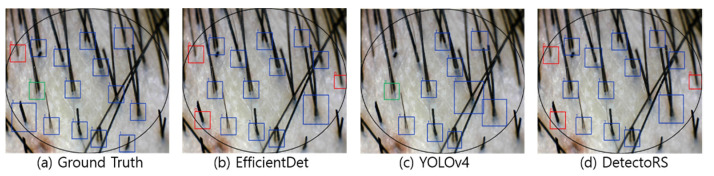
Visualization of detection results in a long hair image.

**Figure 6:**
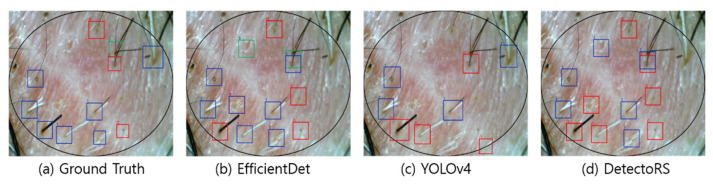
Visualization of detection results in a white hair image.

**Figure 7:**
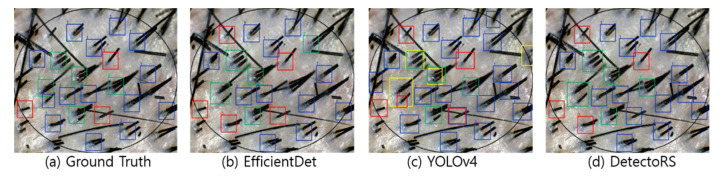
Visualization of detection results in an image with a large number of short and long hairs.

Figure 4, Figure 5, Figure 6 and Figure 7 visualize the detection results according to the shape of the hair. In the visualization image, the color (Class 0: red; Class 1: blue; Class 2: green; Class 3: yellow) is expressed according to the number of hair follicles (Class 0: one; Class 1: two; Class 2: three; Class 3: four or more). In this experiment, to minimize false detection of the blurred boundary area, an ellipse was drawn based on the center of the input image to compare the detection performance only for the hair follicles inside. However, as shown in the figures, because the number of hair follicles existing outside the ellipse was relatively small, it did not appear to have a significant effect on the evaluation of the overall performance.

Figure 4 shows the results of hair follicle detection in a hair scalp RGB image with many short strands of hair. All three models showed accurate follicle detection and classification for Classes 1 and 2. Moreover, YOLOv4 showed the performance closest to the ground truth among the models. Although all three models failed to detect some hair follicles, it should be noted that the hair follicles were located at the elliptical boundary and had a relatively blurred image. However, in normal hair scalp images without hair follicles obscured by foreign substances or other hair, the detection accuracy was relatively high, suggesting the feasibility of automating HDM.

Figure 5 shows the results of hair follicle detection in a hair scalp RGB image containing many long strands of hair. Compared with Figure 3, the length of the hair is relatively long, with a greater number of hair strands. Despite some of the hair follicles being obscured by other long hairs, all three models demonstrated accurate detection and classification performance. Furthermore, both EfficientDet and DetectoRS were able to detect relatively blurry hair follicles that were located at the inner boundary of the ellipse. The detection performance of the three networks in Figure 4 was quite encouraging, considering that the long strands of hair in the image hindered the feature extraction by obscuring the hair follicles.

As shown in Figure 6, in cases with many strands of white hair or few strands of hair, the detection performance of all three models was degraded compared to other cases. As for the low detection performance for white hair, the white hair strands may have been considered a foreign substance present in the scalp, resulting in a failure in the classification of hair follicles. YOLOv4 detected empty follicles accurately, whereas both EfficientDet and DetectoRS classified empty follicles as Class 1. Furthermore, when both white and black hair strands were present in the same follicle, neither EfficientDet nor DetectoRS detected such a follicle successfully.

The detection and classification results are shown in Figure 7 for the hair scalp RGB image, from which both short and long hair strands exit. In some cases, YOLOv4 duplicated the detection of one hair follicle with different classes. However, all three models showed detection performance accurate enough to find hair follicles that did not exist in the ground truth, and it was expected that performance would be improved by using large-scale datasets containing accurate annotation information in the future.

Finally, Table 4 shows the performance of the three detection models with and without data augmentation in terms of mAP(50:95).

**Figure 8:**
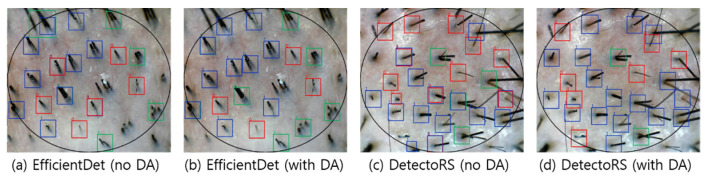
Visualization of detection results with and without data augmentation (DA: data augmentation).

The performance boosts by the data augmentation were clearly visible in the case of EfficientDet and DetectoRS, whereas in YOLOv4, there was no significant difference in performance as a result of applying data augmentation. This result appears to have been caused by the Mosaic data augmentation employed by YOLOv4. The Mosaic data augmentation combines four training images into a synthetic image, allowing the model to learn how to detect small objects. According to the visualization results, EfficientDet showed an improvement in the classification of Class 1 and Class 2 with data augmentation, whereas the redundant detection of the same hair follicles was reduced for DetectoRS (see Figure 8).

## 5. Discussion

In this study, the feasibility of automated HDM was evaluated using state-of-the-art deep-learning-based object detection technology (specifically, EfficientDet, YOLOv4, and DetectoRS). For training and validation of the deep learning models, 4492 enlarged hair scalp RGB images from 817 patients obtained from real-world clinical practice were used. Based on the experimental results, YOLOv4 achieved the highest mAP of 58.67. Compared with EfficientDet and DetectoRS, YOLOv4 had a lower rate of redundant detection while demonstrating better classification of all four classes. However, for hair follicles of Class 3, all three detection models showed poor performance. In particular, there were many false detections where a hair follicle with four strands of hair was detected as two hair follicles, each with two strands of hair, or two hair follicles, one with a single strand of hair and the other with three strands of hair. One potential reason for this is that the images of Class 3 contain features similar to those in the images of Class 1 or Class 2. In addition, the class imbalance between Class 3 and the other classes may have led to poor performance. To address these problems, it is necessary to develop sophisticated feature representation algorithms and/or train the models using a large number of sample images containing hair follicles with four or more hairs.

Future work will be conducted in several areas. In general, deep learning models are able to recognize more patterns with the availability of more training data. Therefore, we plan to utilize additional data from multiple institutions for model training and testing. In addition, future work should validate whether similar results can be obtained using other state-of-the-art deep learning models.

In conclusion, the experimental results suggest that a deep-learning-based algorithm could provide an acceptable level of accuracy for automated HDM with a sufficient number of training datasets available.

## Figures and Tables

**Table 1:** Demographic information of the dataset.

Classification	Information
The number of data samples	Male (4492)/Female (0)
Mean Age	42 years

**Table 2:** Hyperparameter settings.

Models	Iterations (Epochs)	Batch Size	Learning Rate	Optimizer	Learning Time (h)
EfficientDet	100	16	1 × 10^−4^	Stochastic Gradient Decent (SGD)	20
YOLOv4	100	32	1 × 10^−3^	Adam	24
DetectoRS	100	16	1 × 10^−4^	SGD	30

**Table 3:** Comparative performance of the deep learning models.

Models	Map	mAP(50)	mAP(75)	Precision	Recall	Accuracy
EfficientDet	31.97	53.45	35.38	71.24	64.09	64.71
YOLOv4	58.67	73.11	60.85	80.75	80.22	75.73
DetectoRS	37.22	58.13	40.64	71.26	71.60	66.36

**Table 4:** Effects of data augmentation.

Models	Without Data Augmentation					With Data Augmentation				
	Class 0	Class 1	Class 2	Class 3	mAP	Class 0	Class 1	Class 2	Class 3	mAP
EfficientDet	25.39	30.61	26.62	16.74	24.39	30.49	40.11	36.62	20.65	31.97
YOLOv4	63.27	70.94	60.75	34.29	57.31	64.21	72.95	61.68	35.82	58.67
DetectoRS	20.41	31.59	23.20	17.65	23.21	34.66	50.92	38.41	24.87	37.22

## Data Availability

The dataset used in this study is released by the National Information Society Agency and is accessible at http://aihub.or.kr (accessed on 12 January 2022).
